# Natural compounds as lactate dehydrogenase inhibitors: potential therapeutics for lactate dehydrogenase inhibitors-related diseases

**DOI:** 10.3389/fphar.2023.1275000

**Published:** 2023-10-17

**Authors:** Jung Ho Han, Eun-Ji Lee, Wonyoung Park, Ki-Tae Ha, Hwan-Suck Chung

**Affiliations:** ^1^ Korean Medicine (KM)-Application Center, Korea Institute of Oriental Medicine (KIOM), Daegu, Republic of Korea; ^2^ Department of Korean Medical Science, School of Korean Medicine, Pusan National University, Yangsan, Republic of Korea; ^3^ Korean Convergence Medical Science Major, KIOM Campus, University of Science and Technology (UST), Daegu, Republic of Korea

**Keywords:** lactate dehydrogenase, natural compound, LDH inhibitor, pharmacological effect, cancer, cardiovascular disease, neurodegenerative disease

## Abstract

Lactate dehydrogenase (LDH) is a crucial enzyme involved in energy metabolism and present in various cells throughout the body. Its diverse physiological functions encompass glycolysis, and its abnormal activity is associated with numerous diseases. Targeting LDH has emerged as a vital approach in drug discovery, leading to the identification of LDH inhibitors among natural compounds, such as polyphenols, alkaloids, and terpenoids. These compounds demonstrate therapeutic potential against LDH-related diseases, including anti-cancer effects. However, challenges concerning limited bioavailability, poor solubility, and potential toxicity must be addressed. Combining natural compounds with LDH inhibitors has led to promising outcomes in preclinical studies. This review highlights the promise of natural compounds as LDH inhibitors for treating cancer, cardiovascular, and neurodegenerative diseases.

## 1 Introduction

LDH is a crucial enzyme in metabolism, catalyzing the interconversion of pyruvate and lactate (Farhana and Lappin, 2022). It plays a vital role in physiological processes, including energy metabolism, glycolysis, and intracellular redox regulation (Kane, 2014). In recent years, there has been an increase recognition of the therapeutic potential of natural compounds in modulating LDH activity and expression and addressing LDH-related diseases (Gao and Chen, 2015; Li et al., 2019b; Forkasiewicz et al., 2020). The regulation of LDH can lead to various effects, including anti-oxidative stress, anti-inflammatory responses, and anti-apoptotic processes (Liu, 1995; Liu et al., 2010; Venkatesan et al., 2015). It can also affect related pathways and downstream signaling associated with the LDH (Zha et al., 2011; Miao et al., 2013; Feng et al., 2018). The involvement of LDH in several diseases, such as cancer, cardiovascular diseases, and neurodegenerative disorders, suggests that natural compounds have a broader therapeutic potential for LDH-related diseases (Liao et al., 2012; Liu et al., 2017; Morandi and Indraccolo, 2017).

Aberrant LDH activity has made the enzyme an attractive target for drug discovery (Varghese et al., 2020). Many LDH inhibitors have been discovered (Rivera et al., 2009), including natural compounds, such as polyphenols, alkaloids, and terpenoids (Gallagher et al., 2017; He et al., 2021; Ramakrishna et al., 2021). Polyphenols, abundant in plants, inhibit LDH activity by binding to its active site, decreasing lactate production (Granchi et al., 2010). Promising polyphenols for treatment of diseases related to abnormal LDH activity, such as cancer, include quercetin, kaempferol, and apigenin (Miean and Mohamed, 2001). Alkaloids, including berberine and magnoflorine, also inhibit LDH by binding to its active site, thereby preventing lactate production (Huang et al., 2009; Kooshki et al., 2022), and exert various pharmacological effects, including anti-cancer, anti-microbial, and anti-inflammatory activities (Aggarwal et al., 2011; Gurung and De, 2017; Reddy et al., 2020). Similarly, terpenoids, such as carnosic acid and artemisinin, inhibit LDH activity (Akinloye et al., 2021) by binding to the enzyme’s active site (Cameron et al., 2004; Hou et al., 2012) and show potential as anti-cancer agents, with their additional pharmacological effects including anti-inflammatory and anti-microbial activities (Salminen et al., 2008; Doughari, 2012).

However, natural compounds face challenges in terms of clinical development owing to limited bioavailability, inadequate solubility, and potential toxicity (Shishir et al., 2019; Yadav et al., 2019; Garcia-Oliveira et al., 2021). Researchers have attempted to enhance their bioavailability while minimizing toxicity using drug delivery systems (Aqil et al., 2013; Ting et al., 2014). Combining LDH inhibitors from natural compounds with synthetic compounds in therapy has shown promise in preclinical studies, suggesting that these compounds could enhance therapeutic effects (Li et al., 2013; Han et al., 2015; Cui et al., 2017).

This review explores the significance of LDH in a range of diseases, including cancer, cardiovascular diseases, and neurodegenerative diseases. It discusses the challenges in developing and using natural LDH inhibitors, their impact on downstream signaling pathways after LDH modulation, the mechanisms of their actions, and potential combination treatments with conventional medications. Natural compounds have the potential to be beneficial therapeutics for LDH-related diseases, and future research opportunities are also discussed.

## 2 Lactate dehydrogenase

LDH is a vital enzyme present in almost all cells, playing an essential role in energy metabolism (Le et al., 2010; Hu et al., 2016). It catalyzes the conversion of lactate to pyruvate and *vice versa*, depending on cellular energy demands (Gladden, 2004; Farhana and Lappin, 2022). This reaction involves the interconversion of cofactors, nicotinamide adenine dinucleotide (NAD^+^) and *ß*-nicotinamide adenine dinucleotide hydrate (NADH), which are essential for energy transfer in living organisms (Rodriguez et al., 2019). LDH operates as a proton donor, with His (193) serving as the proton donor, Arg (99) as the coenzyme, Asn(138) as the hydrogen bond donor, and Arg (106), Arg (169), and Thr (248) as substrate binding residues (Holmes and Goldberg, 2009).

**FIGURE 1:**
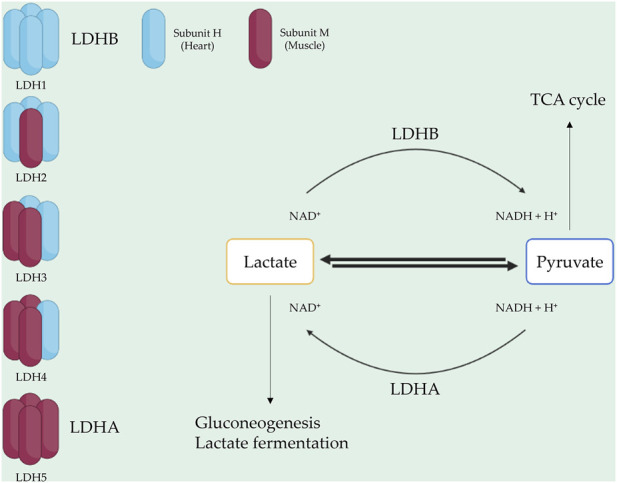
LDH composition and function. LDH is a tetrameric enzyme with five isotypes. Each isotype consists of two subunit types: H (heart) and M (muscle). LDH1 and LDH5, represented by LDHB and LDHA, play roles in converting lactate to pyruvate and pyruvate to lactate, respectively.

LDH consists of two subunits: LDH-heart (H) and LDH-muscle (M), encoded by the genes *LDHA* and *LDHB*, respectively (Al-Jassabi, 2002). The H subunit is predominant in the brain and heart, whereas the M subunit is found in skeletal muscle tissues (Woodford et al., 2019). Two popular isoforms of LDH exist, resulting in five isotype enzymes: LDH1 (H4), LDH2 (H3M1), LDH3 (H2M2), LDH4 (H1M3), and LDH5 (M4) (Al-Jassabi, 2002). LDHA, also known as LDH5, is highly expressed in skeletal muscle and catalyzes pyruvate and NADH to lactate and NAD+, with this reaction being crucial for aerobic glycolysis metabolism in skeletal muscle (Kane, 2014). Conversely, LDHB, also known as LDH1, is abundant in the heart and brain, where it converts lactate and NAD + to pyruvate and NADH (Read et al., 2001a; Doherty and Cleveland, 2013). LDH2, LDH3, and LDH4 are found in lung tissue, bone marrow, and the pancreas (Aliberti et al., 1997; Ben et al., 2007). Each isotype enzyme exhibits intermediate activity levels from LDHA to LDHB, depending on tissue metabolic needs (Johari et al., 2018) (Figure 1).

LDH activity is associated with various diseases, including cancer, cardiovascular diseases, and neurodegenerative disorders (Dhanasekaran and Ren, 2005; Roychoudhury et al., 2021). Dysregulated LDH activity contributes significantly to cancer development, promoting the Warburg effect (Chen et al., 2007), which involves increased glucose uptake and lactate production, even in the presence of oxygen, to meet the energy demands of rapidly proliferating cancer cells (Warburg and Minami, 1923; Dai et al., 2016b). LDHA overexpression favors pyruvate to lactate conversion, leading to tumor microenvironment acidification and aiding cancer progression and metastasis (Vander Heiden et al., 2009). Abnormal LDH activity is also observed in other diseases. For example, increased LDH activity has been reported in cardiovascular diseases, such as myocardial infarction and heart failure, reflecting cardiac tissue damage and necrosis (Ndrepepa, 2021). Neurodegenerative diseases, including Alzheimer’s disease and Parkinson’s disease, are associated with elevated LDH activity, potentially reflecting neuronal damage and inflammation (Fahrig et al., 2005; Di Domenico et al., 2017).

Given the critical role of LDH in disease development, targeting the enzyme has become an essential strategy for drug discovery. Numerous natural compounds, including polyphenols, alkaloids, and terpenoids, have shown promising results as potential LDH inhibitors for disease treatment.

## 3 LDH inhibitors: types, mechanisms, and therapeutic applications

### 3.1 Categorization of LDH inhibitors

We now explore LDH inhibitors in more depth, classifying them based on their chemical structures and modes of action into two categories: small-molecule inhibitors and RNA-based inhibitors.

#### 3.1.1 Small-molecule inhibitors

**FIGURE 2:**
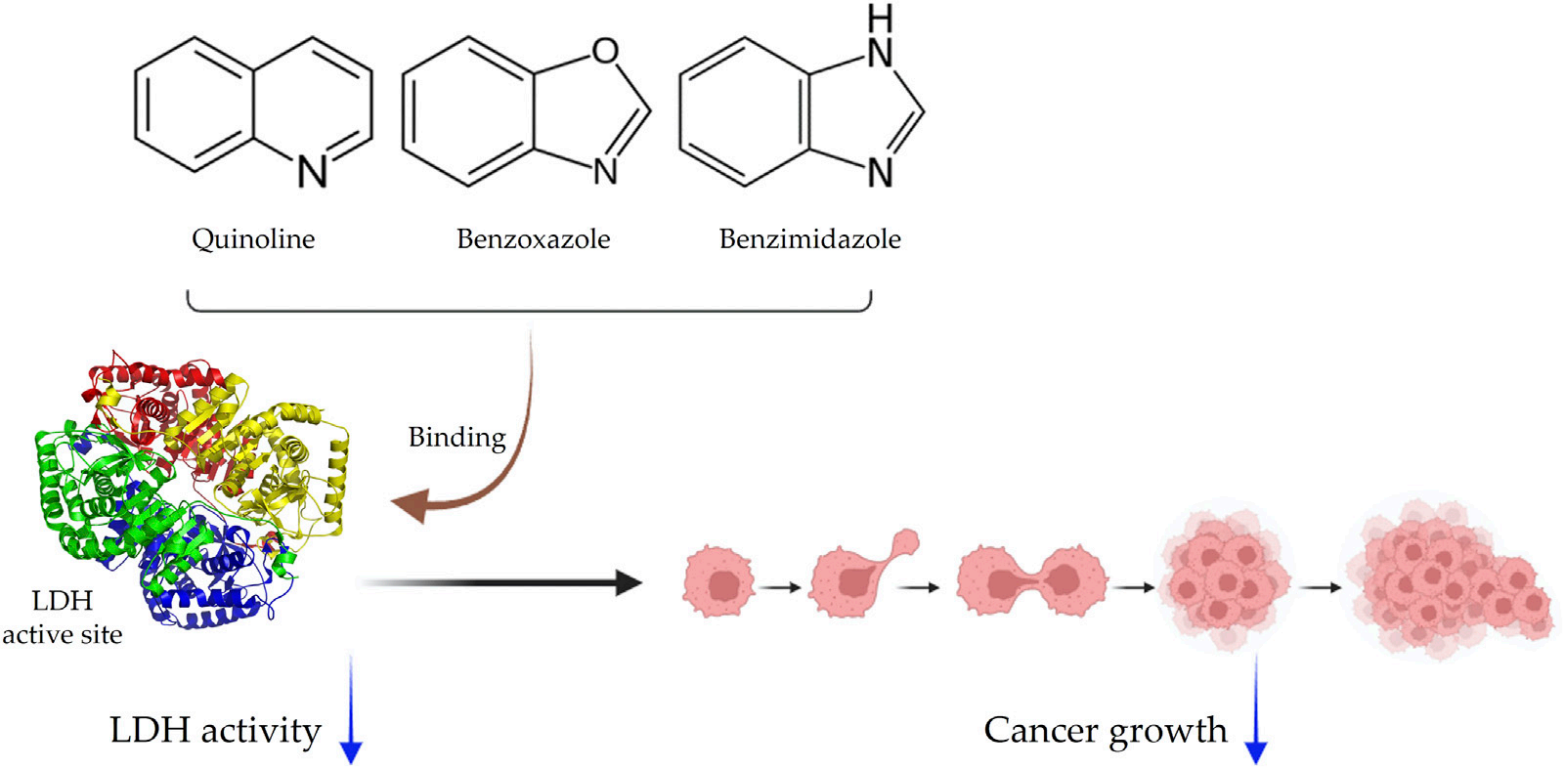
Schematic representation of small-molecule inhibitors. Inhibitors are categorized as quinolines, benzoxazoles, and benzimidazoles. They bind to the LDH active site and effectively inhibit cancer growth.

Small-molecule inhibitors, compounds with low molecular weight, are often effective LDH inhibitors that can penetrate cell membranes and bind to the active site of the enzyme, hindering its function (Vander Heiden et al., 2010; Granchi et al., 2013). These inhibitors can be further categorized based on their chemical composition, with main subcategories including quinoline-based inhibitors with quinoline rings, benzoxazole-based inhibitors with benzoxazole rings, and benzimidazole-based inhibitors with benzimidazole rings (Holmes et al., 2006; Madapa et al., 2008; Kanwal et al., 2018). Studies have shown that these inhibitors effectively reduce LDH activity in cancerous cells and possess anti-cancer properties both *in vitro* and *in vivo* (Granchi and Minutolo, 2012; Piekuś-Słomka et al., 2019; Zhou et al., 2020). Some specific LDHA inhibitors, such as FX-11 (a benzoxazole-based inhibitor) and Compound 3a (a quinoline-based inhibitor), have shown selective inhibition of cancer cell growth. Another effective class of inhibitors is the benzimidazole anthelmintics (Miao et al., 2013; Rani and Kumar, 2016; Son et al., 2020). Depending on their chemical structure, small-molecule inhibitors may selectively target LDHA or LDHB isoforms (Fiume et al., 2014) (Figure 2).

#### 3.1.2 RNA-based inhibitors

A novel group of LDH inhibitors is RNA-based, with these inhibitors specifically hindering the expression of LDH-related enzymes (Liu et al., 2021). RNA-based inhibitors are categorized into two types based on their underlying mechanisms: RNA interference (RNAi) and antisense oligonucleotides (ASOs) (Post et al., 2019; Maruyama and Yokota, 2020).

RNAi occurs naturally, where small interfering RNAs (siRNAs) degrade mRNA, leading to gene silencing (McManus and Sharp, 2002). By pairing with complementary mRNA sequences, double-stranded RNA molecules trigger the RNA-induced silencing complex, breaking down the mRNA (Sontheimer, 2005). RNAi-based LDH inhibitors target mRNA sequences responsible for encoding LDH, resulting in its downregulation and decreased LDH activity (Yang and Zhang, 2012).

ASOs inhibit protein translation by binding to complementary mRNA sequences. ASOs are short single-stranded RNA molecules with effective inhibition capabilities (Ding and Lawrence, 2001). LDH-specific ASOs can be designed to target mRNA sequences encoding the LDH enzyme, leading to decreased expression and inhibition of its activity (Manjunath et al., 2022). RNA-based inhibitors offer advantages over small-molecule inhibitors (Blom et al., 2022). For instance, they can be designed for highly selective LDH inhibition by targeting specific mRNA sequences (Manjunath et al., 2022). Furthermore, RNA-based inhibitors can exhibit longer action time for inhibition and can be administered using viral vectors and lipid nanoparticles (Mogler and Kamrud, 2015; Bajan and Hutvagner, 2020; Aldosari et al., 2021).

### 3.2 Mechanisms of action

Various natural compounds can inhibit LDH activity through different mechanisms. The most common approach involves direct binding to the enzyme’s active site, leading to the inhibition of pyruvate conversion to lactate (Conners et al., 2005). Polyphenols, such as quercetin and epigallocatechin gallate (EGCG), can bind to the active site of LDH, reducing its activity levels (Gradišar et al., 2007). Additionally, some natural compounds modulate other components involved with LDH, including lactate transporters or mitochondrial enzymes. For instance, rosmarinic acid affects the lactate transporters of cancer cells (Marin-Hernandez et al., 2009; Cerella et al., 2013; Ma et al., 2018).

Another mechanism underlying inhibition of LDH by natural compounds is the regulation of LDH gene expression. Various compounds, such as curcumin, resveratrol, and quercetin, can inhibit LDH expression by reducing LDH gene transcription or promoting LDH protein degradation (Yang et al., 2018; Reyes-Farias and Carrasco-Pozo, 2019; Soni et al., 2020). In contrast to small-molecule inhibitors, RNA-based inhibitors target LDH expression by interfering with its mRNA. As discussed earlier, RNAi and ASOs are commonly used for inhibiting LDH expression, which is achieved at the mRNA level (Wood et al., 2019; Bockstahler et al., 2022).

#### 3.2.1 Inhibition of LDH enzyme activity

As low-molecular-weight substances, small-molecule inhibitors, often referred to as drug-like molecules, can easily penetrate cells and interact with their target enzymes (Makley and Gestwicki, 2013). These inhibitors effectively block the enzyme’s catalytic activity by competing with the substrate for binding to the active site (Copeland et al., 2007). Based on their mode of action, small-molecule inhibitors can be further categorized as reversible or irreversible inhibitors (Roskoski Jr, 2016). Reversible inhibitors bind to the enzyme’s active site noncovalently and can be displaced by excess substrate, whereas irreversible inhibitors bind to the enzyme covalently, leading to permanent inactivation (Purich, 2010) Examples of small-molecule LDH inhibitors include FX11, which selectively inhibits LDHA, and gossypol, which inhibits both LDHA and LDHB (Conners et al., 2005; Granchi et al., 2011).

#### 3.2.2 Inhibition of LDH enzyme expression

RNA-based inhibitors are employed to inhibit LDH enzyme expression, through RNAi and ASOs (Liu et al., 2021). These inhibitors induce mRNA degradation through siRNAs and inhibit mRNA translation via complementary sequences with ASOs (Ding and Lawrence, 2001; Sontheimer, 2005). RNA-based inhibitors exhibit high specificity, selectively targeting enzymes and effectively inhibiting LDH. Moreover, they can be efficiently delivered to the target using lipid nanoparticles or viral vectors (Mogler and Kamrud, 2015; Younis et al., 2021). However, some limitations, such as off-target effects and limited delivery to specific tissues or cell types, exist (Sibley et al., 2010; Lu and Thum, 2019).

### 3.3 Therapeutic applications

Several studies have demonstrated the potential therapeutic effects of LDH inhibitors in diseases associated with abnormal LDH activity (Granchi et al., 2010; Valvona et al., 2016). For instance, preclinical studies have shown that LDH inhibitors can suppress tumor growth both as a monotreatment and in combination with other cancer therapies, such as chemotherapy and radiation therapy (Capula et al., 2019; Qiao et al., 2021). LDH inhibitors have also been investigated as potential therapeutics in cardiovascular diseases (Ji et al., 2021), as well as for their effects on Alzheimer’s disease and Parkinson’s disease (Newington et al., 2013; Acharya et al., 2019).

#### 3.3.1 Cancer

Cancer cells often exhibit high levels of LDH activity, which supports uncontrolled cell growth and migration, (Gallo et al., 2015), especially under hypoxic conditions (Han et al., 2021). Consequently, LDH inhibition has emerged as a promising therapeutic approach for cancer therapy (Pi et al., 2022). In preclinical studies, LDH inhibition has resulted in anti-cancer effects both under monotreatment and combination therapy with chemotherapy and radiation therapy (Abdel-Wahab et al., 2019). For example, the LDH inhibitor FX-11 has been shown to reduce tumor growth and enhance the efficacy of chemotherapy (Fantin et al., 2006). As FX-11 inhibits LDH activity, it promotes a shift toward oxidative phosphorylation and impaired cancer cell growth and survival (Le et al., 2010). LDHA inhibition by oxamate resulted in the accumulation of reactive oxygen species (ROS) and depletion of adenosine triphosphate (ATP), leading to increased sensitivity to radiotherapy in A549 and H1975 cancer cells (Yang et al., 2021).

Silibinin, a natural compound found in milk thistle, has also been proven to inhibit LDH and reduce tumor growth in various cancer cell lines (Milić et al., 2013). In a chemically induced skin cancer model in mice, silibinin reduced the expression of the tumor necrosis factor-α endogenous promoter (Zhao et al., 1999). In hepatocellular carcinoma in rats, it decreased levels of malondialdehyde (MDA)-DNA (Ramakrishnan et al., 2007). Silibinin’s potential extends to human ovarian cancer, where it inhibits tumor growth by downregulating VEGFR receptor 3 (Gallo et al., 2003). In cervical cancer, silibinin induces apoptosis through MAPK (mitogen activated protein kinase) activation, characterized by chromatin condensation and nuclear fragmentation (Huang et al., 2005). Moreover, silibinin’s pre-treatment reduces the phosphorylation of signal transducer and activator of transcription 1 (STAT1) and signal transducer and activator of transcription 3 (STAT3) induced by cytokines responsible for the proliferation of A549 human lung cancer cells *in vitro*. Silibinin also inhibits the AP-1 transcription factor of DNA and blocks the MAPK cascade (Chittezhath et al., 2008). In prostate cancer, it downregulates epidermal growth factor receptor (EGFR) signaling, leading to cell cycle arrest and reduced expression of tumor growth factor (TGF-α) (Singh and Agarwal, 2004). Silibinin’s effects extend to oral cancer, where it decreases cell viability by inhibiting akt phosphorylation, resulting in apoptosis (Su et al., 2013). Additionally, Silibinin shows potential in treating gastric cancer by inhibiting the growth of SGC-7901 cells, lowering p34cdc2 levels, and increasing the expression of p53 and p21 (Zhang et al., 2013b). Lastly, in colon cancer, silibinin induces dose-dependent cell cycle arrest, affects autocrine TGF-α secretion, and inhibits EGFR expression (Hogan et al., 2007). It also inhibited oxidative damage caused by lung and brain sepsis by balancing the oxidative status and modulating inflammatory mediators (Toklu et al., 2008). It induces apoptosis, suppresses angiogenesis, and decreases the expression of hypoxia-inducible factors (Sameri et al., 2021).

Similarly, the LDH inhibitor galloflavin is known to enhance efficacy in radiation therapy in preclinical studies (Kozal et al., 2021), which it achieves by sensitizing cancer cells to radiation-induced DNA damage, leading to enhanced cancer cell death (Fiume et al., 2013). Galloflavin has demonstrated its efficacy in inhibiting cell growth in endometrial cancer cell lines and primary cultures of human endometrial cancer. It achieves this by engaging with multiple signaling pathways that regulate crucial aspects such as metabolism, cell cycle progression, apoptosis, cellular stress responses, and metastasis (Han et al., 2015). Moreover, inhibition of LDH by galloflavin can exert a growth-inhibitory effect in breast cancer cells. This anti-proliferative effect may result from various mechanisms, including the downregulation of survival signaling pathways and the induction of oxidative stress states (Farabegoli et al., 2012). In Burkitt lymphoma cells, the LDH inhibitor galloflavin reduces cellular NAD levels and leads to the inhibition of sirtuin-1. As confirmed in previous studies, sirtuin-1 inhibition leads to a reduction in MYC protein levels, depriving Burkitt lymphoma cells of a crucial survival signal (Vettraino et al., 2013). Furthermore, in pancreatic cancer cells, a combination of galloflavin and metformin has been found to enhance their effectiveness in inhibiting the proliferation of cancer cells (Wendt et al., 2020).

#### 3.3.2 Cardiovascular diseases

LDH has been implicated in the pathogenesis of various cardiovascular diseases, including cardiac failure and ischemia-reperfusion injury (Kotlyar et al., 2010). Elevated LDH levels under these conditions indicate cellular damage and impaired metabolism (Ait-Aissa et al., 2019). LDH inhibitors have exhibited protective effects against ischemia-reperfusion injury and heart failure in preclinical studies (Zhou et al., 2015). For example, in a rat model simulating myocardial ischemia-reperfusion injury, the LDH inhibitor galloflavin showed the potential to decrease infarct size and improve cardiac function (Gandhi et al., 2022). EGCG has also been shown to inhibit LDH activity, leading to reduced lactate production and cardioprotective effects (Eng et al., 2018). Additionally, S-allylcysteine treatment was shown to improve cardiac function in rats while decreasing oxidative stress and mitochondrial permeability (Aziz et al., 2021). Allicin, found in garlic, demonstrated a significant vasodilating effect on coronary arteries, leading to increased coronary blood flow in the experimental group both before ischemia and during reperfusion. This effect is attributed to a reduced concentration of LDH release (Adegbola et al., 2017). Similarly, curcumin also exhibits cardioprotective effects, as demonstrated in a rat model of acute myocardial infarction by a reduction in serum LDH levels by curcumin intake (Rahnavard et al., 2019).

#### 3.3.3 Neurodegenerative diseases

Increased LDH activity has been detected in the brains of patients with Alzheimer’s (Bigl et al., 1999). The effects of *Lycium barbarum* extract on cell models of Alzheimer’s disease have been investigated, with a significant reduction in the release of LDH and a dose-dependent neuroprotective effect observed (Yu et al., 2005). This extract has also demonstrated effectiveness in treating Alzheimer’s disease by protecting against neurotoxicity caused by beta-amyloid peptides (Ho et al., 2007). EGCG has demonstrated enhanced effectiveness in neuroprotection by significantly reducing lactate dehydrogenase release in a cell model of Parkinson’s disease. Furthermore, Western blot analysis indicated that Akt might be one of the specific signaling pathways stimulated by EGCG in the context of neuroprotection (Chao et al., 2010). Overexpression and abnormal accumulation of *a*-synuclein are associated with Parkinson’s disease and result in increased intracellular ROS, causing mitochondrial dysfunction and oxidative damage in a Parkinson’s disease model. The use of curcumin demonstrated a reduction in LDH release, alleviating αS-induced toxicity, lowering ROS levels, and providing protection to cells against apoptosis (Wang et al., 2010).

## 4 Natural compounds as LDH inhibitors: Polyphenols, alkaloids, terpenoids and sulfur-containing agent

### 4.1 Polyphenols and cancer

Flavonoids, a group of polyphenols abundant in fruit, vegetables, and medicinal plants, function as LDH inhibitors (Huang et al., 2009). Studies have shown that the flavonoids curcumin and quercetin inhibit LDH activity and reduce lactate synthesis in cancer cells (Maurya and Vinayak, 2015; Unlu et al., 2016; Reyes-Farias and Carrasco-Pozo, 2019). Curcumin treatment reduces LDHA expression in human colorectal cancer cells, leading to decreased lactate production and cellular proliferation (Wang et al., 2015b). Quercetin, found in several foods, including apples and onions (Kopustinskiene et al., 2020), reduces LDHA activity and triggers apoptosis in cancer cells, effectively inhibiting cellular glycolysis by reducing LDHA expression, thereby suppressing lactic acid generation and glucose uptake (Jia et al., 2018). Kaempferol, found in tea and various fruit, downregulates LDHA expression in human breast cancer cells through inhibition of STAT3 activity (Narayan and Kumar, 2014). Galloflavin binds to the NADH-binding site in LDHA, inhibiting its ability to bind to single-stranded DNA and suppressing colorectal cancer growth (Fiume et al., 2013). Moreover, galloflavin has been shown to completely inhibit both LDHA and LDHB (Manerba et al., 2012). EGCG, the primary flavanol in green tea, inhibits LDHA and exhibits anti-cancer activity in pancreatic cancer cells, and it significantly slows the growth of breast cancer cells, triggering apoptosis through its action as an LDHA inhibitor (Wang et al., 2015a; Gao and Chen, 2015). Combining catechin, epicatechin, and gallocatechin with epigallocatechin enhanced the inhibitory effect on LDHA (Cheng et al., 2020). Apigenin reduces LDHA mRNA expression in HepG2 cells, a human hepatocellular carcinoma cell line (Korga et al., 2019). Although the precise mechanism underlying inhibition of LDH by flavonoids is not fully understood, it may involve direct enzyme binding, gene expression modulation, or protein stability regulation (Bader et al., 2015; Yao et al., 2022). Luteolin acts as an LDH inhibitor and has been found to bind effectively to the active pocket residues of LDH (Li et al., 2021b). Additionally, luteolin 7-O-β-d-glucoside, found in Phlomis kurdica, non-specifically inhibits both LDH-1 and LDH-5 (Bader et al., 2015).

### 4.2 Polyphenols and cardiovascular diseases

Flavonoids have been shown to exert beneficial effects on cardiovascular health, preventing atherosclerosis, hypertension, and myocardial infarction (Stangl et al., 2006). Quercetin treatment was found to significantly decrease infarct size and improve cardiac function in rats with myocardial infarction (Zaafan et al., 2013), with quercetin’s capacity to lower oxidative stress, inflammation, and apoptosis in the heart considered possible causes of its cardioprotective effects.

Hesperidin, found in citrus fruits, exhibits anti-hypertensive and anti-atherosclerotic properties (Mahmoud et al., 2019). It has been shown to lower blood pressure and enhance endothelial function in hypertensive rats (Morand et al., 2011), and it prevents atherosclerotic plaque formation in apolipoprotein E–knockout mice (Sugasawa et al., 2019). Catechin in tea and resveratrol in red wine also improve cardiovascular health (Gross, 2004), with the former regulating lipid metabolism and the latter reducing inflammation, oxidative stress, and platelet aggregation (Chen et al., 2016). These mechanisms contribute to improved cardiovascular disease outcomes (Gresele et al., 2011).

### 4.3 Polyphenols and neurodegenerative diseases

Flavonoids have been extensively studied for their potential in treating neurodegenerative diseases owing to their anti-inflammatory, anti-oxidant, and neuroprotective properties (Spagnuolo et al., 2018). In animal models of neurodegenerative diseases, including Alzheimer’s disease, Parkinson’s disease, and Huntington’s disease, flavonoids prevent neurodegeneration and cognitive decline (Solanki et al., 2015) by inhibiting oxidative stress, inflammation, and protein misfolding, and modulating the signaling pathways involved in cell survival, synaptic plasticity, and neurogenesis (Khan et al., 2020; Numakawa and Odaka, 2021). Some flavonoids may also protect against metabolic dysregulation in neurodegenerative processes by inhibiting LDHA activity in the brain (Zhang et al., 2015a). Additionally, flavonoids have been reported to alleviate brain damage caused by ischemia and reperfusion through LDH inhibition and anti-oxidant effects (Dong et al., 2013). The flavonoid oroxylin A reduces LDH expression (Sajeev et al., 2022) and shows potential for preventing and treating neurological diseases (Lu et al., 2016).

### 4.4 Alkaloids and cancer

Alkaloids, nitrogen-containing compounds widely distributed in the plant kingdom (Roy, 2017), have been recognized for their potential in LDH inhibition and drug discovery (Khazir et al., 2013). Berberine, an isoquinoline alkaloid found in plants, including goldenseal and barberry, possesses anti-bacterial and anti-inflammatory properties, making it a valuable component in Chinese medicine (Imanshahidi and Hosseinzadeh, 2008). Berberine is known to exhibit anti-cancer activity through inhibition of LDHA activity and reduction of lactate production in cancer cells (Tan et al., 2015). In mouse models of breast, colon, and lung cancer, berberine has demonstrated significant anti-cancer effects, inhibiting tumor growth and reducing lactate production (Sun et al., 2009; Mao et al., 2018). Moreover, berberine has shown the ability to suppress LDHA activity, inhibiting pancreatic cancer cell proliferation (Cheng et al., 2021). Papaverine, an isoquinoline-type alkaloid reported to inhibit LDHA, is currently undergoing clinical trials as a radiosensitizer aimed at reducing tumor hypoxia and enhancing the radiotherapy response in A549 non-small cell lung cancer cell (NSCLC) and EO771 breast cancer xenografts (Kapp and Whiteley, 1991; Benej et al., 2018).

### 4.5 Alkaloids and cardiovascular diseases

Alkaloids, such as berberine, vincamine, rutaecarpine, chelerythrine, and matrine, have been investigated for their therapeutic potential in various cardiovascular diseases (Yamamoto et al., 2001; Jia and Hu, 2010; Zhang and Yan, 2020; Zhang et al., 2021b; Cai et al., 2021). Berberine has been found to inhibit LDH activity in H9c2 cardiomyocytes, providing protection against ischemia/reperfusion injury (Zhu et al., 2020). Berberine treatment has also been shown to decrease lactate production and increase ATP production in cardiomyocytes, improving cellular energy metabolism through LDH activity inhibition (Lv et al., 2012).

Leonurine, derived from the Lamiaceae family, exhibits cardioprotective effects by decreasing LDH activity and exerting anti-oxidative activity (Liu et al., 2010). Additionally, rutaecarpine, chelerythrine, and matrine have been shown to inhibit LDH levels (Bao et al., 2011; Chen and Huang, 2012; Wu et al., 2022). Of these, rutaecarpine confers protection against myocardial cell injury by inhibiting the NADPH oxidase–ROS pathway (Tian et al., 2019).

### 4.6 Alkaloids and neurodegenerative diseases

In neurological diseases, LDH can serve as a marker for cell damage or death (Adan et al., 2016). Conditions such as stroke, traumatic brain injury, or neurodegenerative disorders can lead to cellular injury or necrosis (Mehta et al., 2013), causing LDH release into the extracellular space (Al Shammari et al., 2015). Elevated LDH levels in the cerebrospinal fluid or blood indicate cellular damage or loss (Fang et al., 2022). Alkaloids derived from Amaryllidaceae species have shown acetylcholinesterase (AChE) inhibitory activity, making them potential candidates for Alzheimer’s disease treatment (Marucci et al., 2021). These alkaloids protect neurons against glutamate-induced damage, reducing apoptotic nuclei and LDH release, indicating reduced cell death and damage (Cortes et al., 2015). These alkaloids may indirectly affect LDH activity through the regulation of acetylcholine levels, which impact cellular metabolism (Kim et al., 2017).

### 4.7 Terpenoids and cancer

Terpenoids, also known as isoprenoids, are a diverse class of chemical compounds found in a wide range of fruits, vegetables, and herbs (Thoppil and Bishayee, 2011). They exhibit numerous biological activities, including anti-cancer properties, (Yang et al., 2020), making them effective against various cancers, such as skin, breast, colon, pancreatic, and prostate cancers. Terpenoids also possess immune-modulating, anti-viral, anti-allergic, and anti-bacterial properties (Thoppil and Bishayee, 2011). Some terpenoids have shown potential for developing anti-cancer drugs as they inhibit LDH activity and reduce lactate production in cancer cells (Kooshki et al., 2022). In patients with idiopathic pulmonary fibrosis, increased levels of LDHA protein and lactate have been associated with reduced lung function (Judge et al., 2018), and gossypol, a terpenoid, has been studied for its potential to decrease the expression of hypoxia-inducible factor 1 alpha (HIF-1α) in lung fibroblast cells (Judge et al., 2017).

Artemisinins, derived from sweet wormwood (*Artemisia annua*), are well-known for their anti-malarial properties and are widely used for malaria treatment (Das, 2012). Dihydroartemisinin, an artemisinin derivative, exerts inhibitory effects on glycolytic metabolism in NSCLC cell lines by suppressing the glucose transporter glucose transporter 1 and impeding glucose absorption (Mi et al., 2015). This compound can also induce perturbations in lactate generation and a concomitant reduction in ATP synthesis (Guerra et al., 2018). Additional experiments have shown that dihydroartemisinin effectively reduces the expression of pyruvate kinase M2 (PKM2) in K562, HepG2, and ESCC cells (Li et al., 2019a).

Limonin, a limonoid present in tangerines, grapefruit, and oranges, exhibits diverse biological functions, including anti-inflammatory and anti-viral properties (Balestrieri et al., 2011; Yang et al., 2014; Gualdani et al., 2016). It has been reported to have anti-tumor activity against breast, liver, colon, and pancreatic cancers (Rahman et al., 2015; Murthy et al., 2021a). Limonin’s inhibitory effect on hexokinase-2 (HK-2) activity was investigated in hepatocellular carcinoma cells, where it effectively suppressed HK-2 activity, leading to decreased cell proliferation and colony formation through reduced glucose consumption and lactate production (Yao et al., 2018). Nimbolide, a limonoid derived from the neem tree (*Azadirachta indica* A. Juss), has demonstrated cytotoxic effects by regulating proliferation, apoptosis, migration, and invasion in various cancer cell lines (Jaiswara and Kumar, 2022).

Oleanolic acid, a natural triterpenoid, is known for its beneficial properties, including anti-inflammatory, anti-oxidant, anti-microbial, hepatoprotective, and anti-cancer activities (Liu, 1995). In endometriosis research, it inhibits LDHA activity in cell lines and induces apoptotic signaling pathways (Cho et al., 2022). Moreover, it suppresses the mTOR signaling pathway and PKM2 production in other breast and prostate cancer cell lines (Liu et al., 2014).

Ursolic acid, another triterpenoid found in various plants, including apple, basil, rosemary, and lavender (Zerin et al., 2016), exhibits various physiological functions, including antibacterial, anti-cancer, anti-diabetic, anti-inflammatory, and anti-oxidant effects (Mlala et al., 2019). For example, it has been shown to reduce LDHA expression in a breast cancer cell line (Wang et al., 2021a). Additionally, betulinic acid, astragalus saponin, and crocetin have been found to suppress LDHA activity and expression, leading to reduced glucose uptake and downregulation of the glycolysis pathway (Kim et al., 2014; Granchi et al., 2017; Guo et al., 2019; Jiao et al., 2019).

### 4.8 Terpenoids and cardiovascular diseases

In one study, the terpenoid ferruginol was found to reduce LDH and creatine kinase MB levels, indicators of doxorubicin-induced tissue damage (Li et al., 2021a). The study revealed that ferruginol mitigated apoptosis progression, as shown in a TUNEL assay in response to doxorubicin. Ferruginol’s cardioprotective action was demonstrated through the preservation of mitochondrial integrity, limitation of ROS-induced heart damage, and attenuation of apoptosis. These effects are likely mediated through the SIRT1 pathway, which regulates mitochondrial biogenesis and fatty acid oxidation.

Thymoquinone, known for its anti-inflammatory, anti-tumor, and analgesic properties (De Sousa, 2011; Sá et al., 2014; Sobral et al., 2014), exhibits anti-oxidant and vascular relaxant effects in experimental models of cardiovascular disease. Thymoquinone administration in mice improved superoxide dismutase activity, reduced interleukin-6 levels, and prevented cardiovascular side effects (Nemmar et al., 2011). In rats with isoproterenol-induced myocardial infarction treated with thymoquinone, dose-related decreases in plasmatic LDH, thiobarbituric acid reactive substances, and glutathione reductase were observed (Randhawa et al., 2013).

Regarding ursolic acid, prominent expression of LDH among serum marker enzymes was observed in myocardial ischemia–induced mice. Following ursolic acid treatment, significant protection against cardiac injury was evident, with a marked reduction in LDH activity (Radhiga et al., 2012).

### 4.9 Terpenoids and neurodegenerative diseases

Derived from *Ginkgo biloba* leaves, Ginkgolide B is a terpenoid diterpene lactone (Iwamoto et al., 2019) known for its anti-inflammatory and neuroprotective properties (Zhang et al., 2011). Ginkgolide B activates the Trk/Ras/MAPK signaling pathway, promoting neurite growth and secretion of brain-derived neurotrophic factors while reducing levels of ROS, LDH, caspase-3, and other proapoptotic factors.

Limonoids have been shown to enhance neuronal differentiation and neurite outgrowth in rat macrophages by activating the PKA/ERK1/2 signaling pathway (Roy and Saraf, 2006), stimulating the secretion of nerve growth factor, and attenuating LDH activity (Zhang et al., 2013a). Through activation of this pathway, limonoids promote neurite outgrowth in rat macrophages, enhancing neuronal differentiation (Gotoh et al., 1990; Yu et al., 2004).

The vibrant orange hue of carrots and sweet potatoes is due to the presence of ß-carotene (Zeb and Mehmood, 2004), a compound that serves as a precursor to vitamin A and is known for its anti-oxidant properties and potential health benefits (Thomas and Oyediran, 2008). ß-carotene plays a protective role in the brain, guarding against the harmful effects of cadmium-induced oxidative stress (Gonzalez-Burgos and Gómez-Serranillos, 2012). It enhances ATPase activity, reduces LDH activity and lipid peroxidation, and contributes to the surge of both enzymatic anti-oxidants, such as glutathione S-transferase and superoxide dismutase, and nonenzymatic anti-oxidants, including glutathione (Park et al., 2011).

*Eucommia ulmoides Oliv*. Bark contains geniposidic acid, one of its active ingredients (Xie et al., 2015). Geniposidic acid not only inhibits LDH but also PARP, cleaved caspase 3, MMPs, and cytochrome C, while increasing the levels of Bcl-2, Bcl-xL, and BDNF (Kwon et al., 2012). These combined effects result in an anti-apoptotic effect and suggest potential applications in the prevention or treatment of neurodegenerative diseases, such as Alzheimer’s disease (Venkatesan et al., 2015).

### 4.10 Sulfur-containing agents and cancer, cardiovascular disease, neurodegenerative disease

Allicin is a bioactive sulfur compound mainly stored in a precursor form in various plant parts. It is known to possess cardioprotective, anti-microbial, cholesterol-lowering, anti-inflammatory, and anti-tumor properties (Catanzaro et al., 2022). In experiments involving the combination treatment of tamoxifen and allicin on Ehrlich ascites carcinoma, both *in vitro* and *in vivo*, LDH levels were reduced, and there was a significant decrease in tumor growth (Suddek, 2014). Furthermore, in an experiment conducted on male Swiss albino mice, it was confirmed that cardiac oxidative damage was reduced when allicin and doxorubicin were administered together. This reduction in oxidative damage was attributed to a decrease in myocardial expression of activated caspase-3 and cyclooxygenase-2 (Abdel-Daim et al., 2017). In Parkinson’s disease, allicin is also known to have a protective effect against nerve damage related to Parkinson’s disease through its inherent antioxidant function and its ability to reduce LDH release (Liu et al., 2015).

**FIGURE 3:**
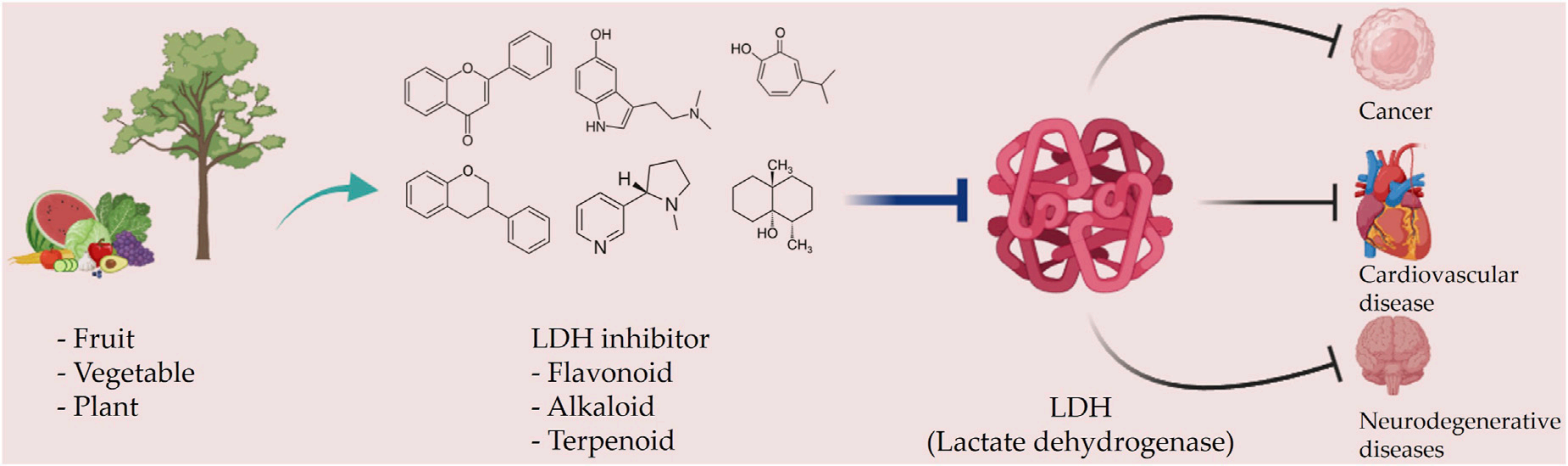
Schematic representation of natural compound LDH inhibitors. Among polyphenols, alkaloids, and terpenoids extracted from plants, certain compounds have therapeutic effects against cancer, cardiovascular diseases, and neurodegenerative diseases through inhibition of LDH.

**FIGURE 4:**
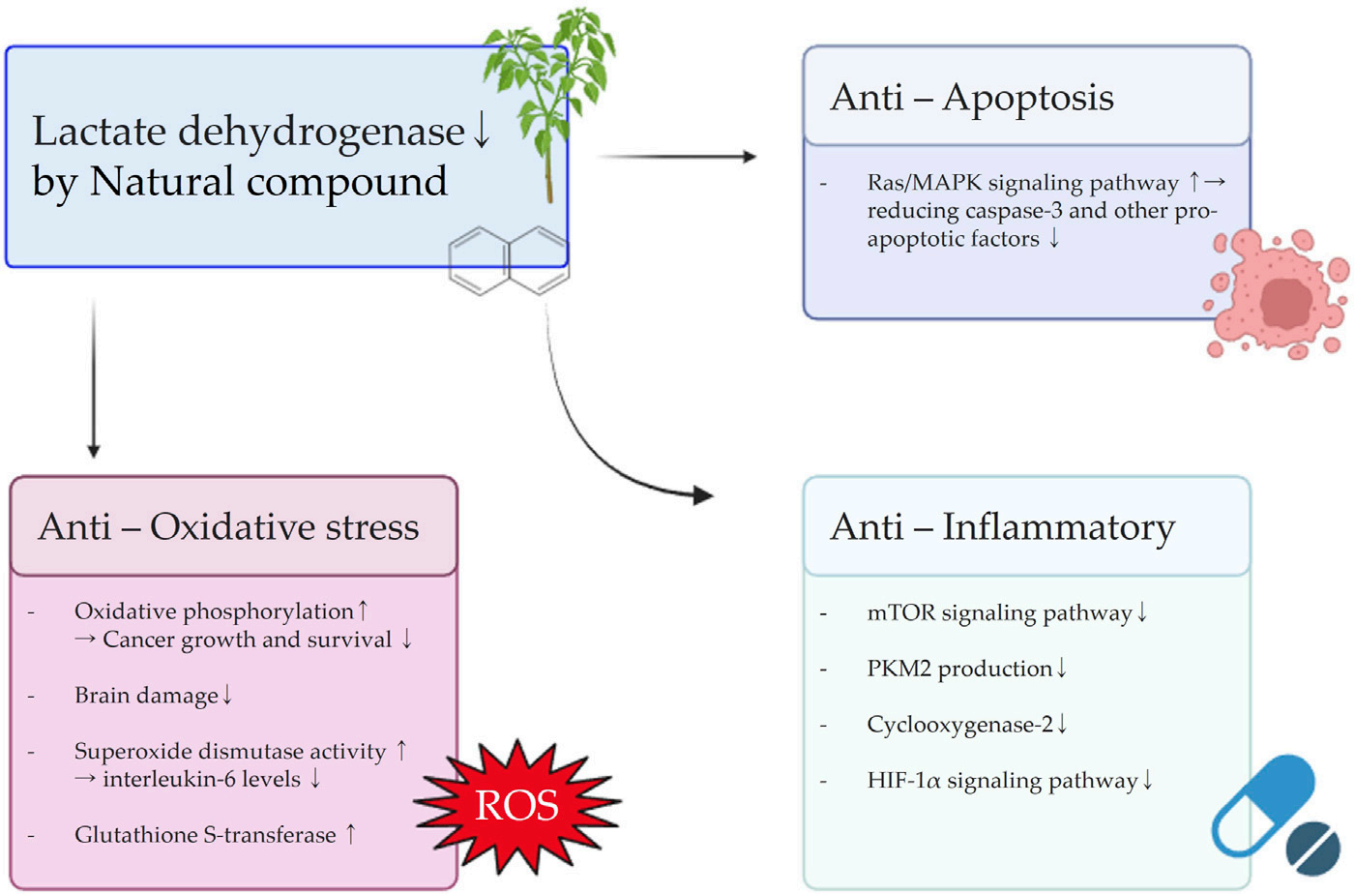
Schematic diagram illustrating the effects of natural compounds on anti-oxidative stress, anti-inflammatory, and anti-apoptosis signals resulting from the inhibition of LDH.

Taurine, an organic compound containing sulfur in its chemical structure, possesses anti-inflammatory, anti-oxidant, and various physiological functions within the cardiovascular, kidney, endocrine, and immune systems (Kim and Cha, 2014). Treatment of the HepG2 cell line, a hepatocellular carcinoma cell line, with taurine resulted in a significant increase in apoptosis-related factors at both the gene and protein levels. Additionally, LDH activity was markedly reduced, indicating the inhibition of glycolysis and cell proliferation (Nabi et al., 2021). Furthermore, taurine has been found to prevent cardiac injury by reducing LDH activity, which is increased by cisplatin (Chowdhury et al., 2016). In neurons, taurine reduced nickel-induced LDH release and mitigated the decrease in ROS production, superoxide dismutase activity, and glutathione concentration, demonstrating its neuroprotective effect through the reduction of oxidative stress (Xu et al., 2015) (Figures 3, 4 and Table 1).

**TABLE 1:** Natural compounds as LDH inhibitors.

Target	Compound	Types of study	Mechanism of action	Ref
LDHA	Curcumin 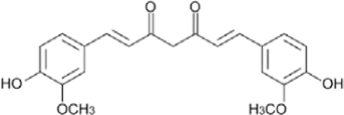	*In vitro* (HCT116, HT29)	Inhibit LDHA expression	Mittal et al. (2020), Fu et al. (2021)
LDHA	Quercetin 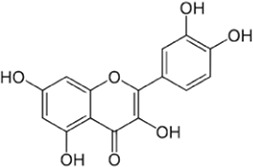	*In vitro* (MCF-7, MDA-MB-231)	Inhibit LDHA activity and mRNA expression	Maurya and Vinayak (2015), Jia et al. (2018), Reyes-Farias and Carrasco-Pozo (2019)
		*In vivo* (DL mice, BALB/c nude mice)	Induce apoptosis	
LDHA	Kaempferol (Polyphenolic components of *Achyranthes aspera*)	*In vivo* (BALB/c mice)	Inhibit LDHA mRNA expression	Narayan and Kumar (2014)
LDHA LDHB	Galloflavin 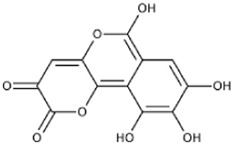	*In vitro* (PLC/PRF/5, SW620)	Inhibit LDHA, LDHB activity	Manerba et al. (2012), Fiume et al. (2013)
LDHA	Epigallocatechin (*Spatholobus suberectus* aqueous extract)	*In vitro* (MCF-7, MDA-MB-231)	Inhibit LDHA activity and expression	Wang et al. (2013)
		*In vivo* (Nude mice)	Accelerated HIF-1α proteasome degradation	
LDHA	Epigallocatechin gallate 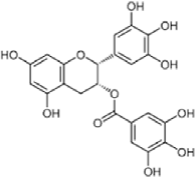	*In vitro* (MIA PaCa-2)	Inhibit LDHA activity and expression	Lu et al. (2015)
LDHA	Apigenin 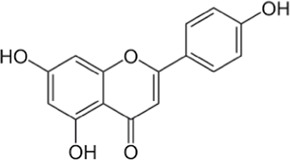	*In vitro* (HepG2)	Inhibit LDHA expression	Korga et al. (2019)
LDHA	Catechin 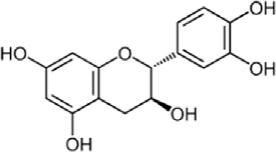	*In vitro* (SNU620, SNU620/5FU)	Inhibit LDHA activity and expression	Han et al. (2021)
LDHA	Oroxylin A 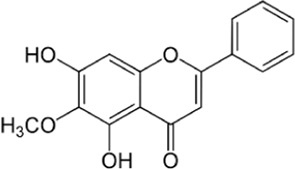	*In vitro* (HepG2)	Inhibit LDHA mRNA expression	Dai et al. (2016a)
LDHA	Berberine 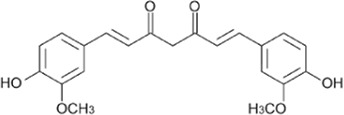	*In vitro* (MCF-7, Hct116, KM12C, pancreatic cancer cell lines)	Inhibit LDHA protein, mRNA expression and activity	Tan et al. (2015), Mao et al. (2018), Cheng et al. (2021)
LDHA	Gossypol 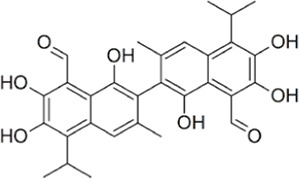	*In vitro* (Mv1Lu)	Inhibit LDHA mRNA expression and activity	Judge et al. (2015), Judge et al. (2017), Judge et al. (2018)
		*In vivo* (C57BL/6 J)		
LDHA	Oleanolic acid 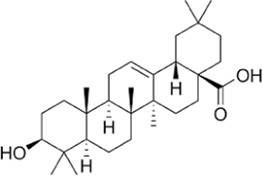	*In vitro* (T-HESCs, 12Z)	Inhibit LDHA activity	Cho et al. (2022)
		*In vivo* (C57BL/6 J)	Induce apoptosis	
LDHA	Ursolic acid 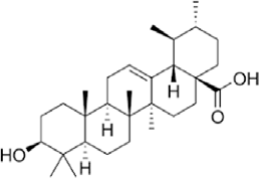	*In vitro* (MCF-7, MDA-MB-231, 4T1, HBL-100, T-HESCs, 12Z)	Inhibit LDHA activity and expression	Wang et al. (2021a), Cho et al. (2022)
		*In vivo* (zebrafish, Balb/c mice, C57BL/6 J mice)	Induce apoptosis	
LDHA	Betulinic acid 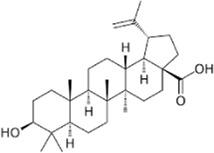	*In vitro* (MCF-7, MDA-MB-231)	Inhibit LDHA expression	Jiao et al. (2019)
		*In vivo* (zebrafish, caveolin-1 knock-out mice)		
LDHA	Astragalus saponin	*In vitro* (HT-29, SW620)	Inhibit LDHA activity and expression	Guo et al. (2019)
		*In vivo* (C57BL/6 J mice)		
LDHA	Crocetin 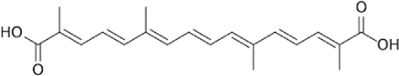	*In vitro* (HeLa)	Inhibit LDHA activity and expression	Kim et al. (2014), Granchi et al. (2017)
LDHA	Limonoid (*Azadirachta indica* A. Juss) 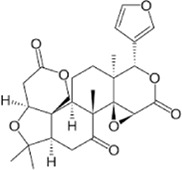	*In vitro* (DL)	Inhibit LDHA protein, mRNA expression	Jaiswara and Kumar (2022)
		*In vivo* (Balb/c mice)		

## 5 Challenges associated with using natural compounds as LDH inhibitors

Owing to their accessibility, diversity, and low toxicity, natural compounds have gained attention as potential disease treatments (David et al., 2015; Rochlani et al., 2017; Mohd Sairazi and Sirajudeen, 2020), with compounds capable of inhibiting LDH activity being of particular interest in disease therapy given its role in energy metabolism (Gallo et al., 2015; Lum et al., 2021; Zhao et al., 2021). However, using natural compounds as LDH inhibitors comes with several challenges that must be addressed before the compounds can become effective treatment options. Some of these challenges are discussed below.

### 5.1 Limited bioavailability

Natural compounds can be rapidly metabolized or excreted from the body, limiting their effectiveness as therapeutics (Fernandes et al., 2014). To address this issue, researchers are exploring various strategies, such as drug delivery systems, chemical modifications, and formulation approaches, to improve the bioavailability of these compounds (Prajapati et al., 2013).

### 5.2 Lack of specificity

Natural compounds often interact with multiple targets in the body, leading to unexpected side effects (David et al., 2015). Some natural compounds may have off-target effects, resulting in side effects, while others may lack specificity toward cancer cells, causing toxicity to normal cells (Mehta et al., 2010). Researchers are developing combination therapies to enhance the specificity of some compounds for cancer cells and minimize side effects (Koehn and Carter, 2005).

### 5.3 Limited understanding of the mechanism of action

The exact mechanism of action for natural compounds as LDH inhibitors remains unclear, hindering their development as therapeutic agents (Augoff et al., 2015). This lack of understanding makes it challenging to enhance their efficacy and minimize potential side effects. Researchers are employing various methods, including computational modeling, biochemical analysis, and proteomics, to elucidate the mechanisms (Lahlou, 2013).

### 5.4 Lack of standardized protocols

The absence of standardized protocols for screening and testing natural compounds as LDH inhibitors leads to inconsistent results. Variations in cell lines and assay conditions can contribute to discrepancies in study outcomes. To address this, many researchers are striving to establish a standardized protocol for screening and evaluating natural compounds (Cos et al., 2006).

### 5.5 Limited commercial interest

The nonpatentable nature of natural compounds has reduced commercial interest in their development as therapeutic agents (Li and Vederas, 2009). Additionally, the high costs associated with clinical trials and regulatory approval pose challenges for investing in natural compound–based drugs (Thomford et al., 2018). To overcome these obstacles, researchers are exploring various business models, such as open-source drug discovery, to incentivize the development of natural compounds as therapeutic agents (Sugumaran, 2012).

## 6 Strategies used to overcome challenges in natural compound development

Several strategies can be employed to address the challenges in developing natural compounds into viable drugs. Some of these strategies are discussed below.

### 6.1 Identification of bioactive compounds

To develop safe and effective drugs, it is essential to identify the major functional compounds in complex natural compounds. Techniques such as high-performance liquid chromatography, gas chromatography–mass spectrometry, and nuclear magnetic resonance, can be used for this purpose (Tsao et al., 2003; Garcia and Barbas, 2011; Wang et al., 2021b). The complexity of some natural compounds makes it challenging to pinpoint the desired therapeutic effect. Utilizing various methods can help identify the principal operational constituent within the complex blend, facilitating the formulation of effective and secure pharmaceuticals.

### 6.2 Optimization of bioactivity

Natural compounds often have low potency and selectivity against the target enzyme due to their low concentrations in their natural sources (Ochoa-Villarreal et al., 2016; Cazzaniga et al., 2021). Optimization of bioactivity can be achieved through structural modification, semi-synthesis, or total synthesis of natural compounds (Prachayasittikul et al., 2015). Structural modification can be applied to enhance the pharmacological properties of natural compounds (Guo, 2017). This process entails altering the compound’s stereochemistry or adding and removing functional groups. For instance, taxol, a natural compound with limited therapeutic potential owing to its low solubility in water (Dordunoo and Burt, 1996), underwent chemical modification to create a more soluble variant known as docetaxel, which has become a popular cancer medication (Hanauske et al., 1992; Fitzpatrick and Wheeler, 2003). Semi-synthesis, which involves analog production through chemical reactions, is another approach for enhancing the pharmacological properties of natural compounds (Lourenco et al., 2012). Although it is not as demanding as total synthesis, semi-synthesis remains effective in improving the compound’s bioactivity. An excellent example of semi-synthesis is the transformation of artemisinin into artesunate, resulting in a more potent variant now used in malaria treatment (White and Olliaro, 1998). Total synthesis the complete chemical synthesis of the natural compound from simple starting materials (Atanasov et al., 2021), represents the most challenging method of bioactivity optimization, but it can also be the most effective. For example, the natural compound shikonin has been completely synthesized, leading to the development of new drugs for the treatment of cancer and other diseases (Andujar et al., 2012; Wang et al., 2012).

### 6.3 Pharmacokinetic optimization

Pharmacokinetic properties, including solubility, stability, bioavailability, and metabolic stability, are crucial considerations in drug development (Sang et al., 2019). Some natural compounds exhibit poor pharmacokinetic properties, which can impede their development as therapeutic agents (Ma et al., 2022). To address this, prodrugs can be used, which are inert compounds that undergo metabolic transformation within the body to generate the active drug (Huttunen et al., 2011). Prodrugs can enhance the stability, solubility, and bioavailability of natural compounds, making them more effective in disease treatment. Additionally, formulation technologies, such as liposomes, nanoparticles, and cyclodextrins, can improve the solubility, stability, and release control of natural compounds, making them easier to administer and more effective (Augustin et al., 2013). Conjugation with suitable carriers, including polyethylene glycol, albumin, and dendrimers, is another approach to optimize pharmacokinetic properties, with this strategy aiming to enhance the overall efficacy of natural compounds as potential therapeutic agents (Gidwani and Vyas, 2015).

### 6.4 Nano-formulations and green synthesis

There are various nano-formulation types, including liposomes, hydrogels, solid lipid nanoparticles, polymeric nanomicelles, dendrimers, chitosan-based nanoparticles, metal nanoparticles, and nanocrystals, which are under investigation for their application with natural compounds (Murthy et al., 2021b). Nano-formulations can safeguard polyphenols against degradation, enhance absorption, and reduce toxicity, making them well-suited for delivering compounds (Khiev et al., 2021). These delivery systems offer advantages such as improved solubility, oral absorption, safety, and bioavailability. Researchers are currently testing the *in vitro* and *in vivo* efficacy of polyphenols like curcumin, quercetin, resveratrol, silybin, luteolin, naringenin, genistein, gossypol, ellagic acid, and hesperidin for treating various diseases (Murthy et al., 2021b).

Curcumin, a polyphenol, exhibits variations in its effects depending on the type and form of nanoparticles and has been extensively studied in various disease models, including malaria, cancer, and cerebral ischemia (Maheshwari et al., 2006; Tsai et al., 2011; Rahimi et al., 2016). The application of these nanoparticle forms has resulted in various effects, including increased solubility and circulation time, enhanced anti-tumor effects and bioavailability, improved anti-oxidative properties, and brain delivery (Liu and Chang, 2011; Liu et al., 2011; Liu et al., 2013). Camptothecin, a natural plant alkaloid, has demonstrated potent anti-tumor activity by targeting intracellular topoisomerase I (Pommier, 2006). The application of nanoformulation has been shown to enhance the efficacy of cancer treatment by addressing limiting factors such as water insolubility (Ghanbari-Movahed et al., 2021). Terpenoids have also been studied for their potential to improve the effectiveness of gastric cancer treatment by increasing anti-cancer and anti-bacterial efficacy through nanoconjugates, and by addressing shortcomings such as target delivery, stability, and half-life (Attri et al., 2023).

The green synthesis of silver nanoparticles from extracts of various plant parts has attracted widespread interest among researchers due to their unique optical and structural properties (Habeeb Rahuman et al., 2022). The green synthesis of nanoparticles is biocompatible and has potential applications in catalysts, anti-bacterial agents, energy harvesting, cancer/gene therapy, and sensing (Rana et al., 2020). Biological methods for nanoparticle synthesis are more economical, easier to implement, have a lower environmental impact, and require fewer processing steps than chemical and physical methods (Kumari et al., 2019). Plants contain polyphenols, flavonoids, alkaloids, and other biomolecules that work synergistically to inhibit oxidative damage to cellular components, leading to the reduction of metal ions into nanoparticles (Mohanpuria et al., 2008; Krishnaraj et al., 2014). Numerous studies have reported the synthesis of gold nanoparticles (AuNPs) using extracts from various plant parts. The putative biomolecules involved in the reduction of gold salts to gold nanoparticles include flavonoids, gingerol, shogaols, gingerone, paradol, catechin, proteins, aromatic amines, and aliphatic amines (Raghunandan et al., 2010; Kumar et al., 2011; Suman et al., 2014). However, the fundamental molecular mechanisms involved in nanoparticle formation are not fully understood, and further studies using natural products are needed.

### 6.5 Target specificity

Natural compounds often possess broad-spectrum activity against multiple targets, posing a challenge in developing specific inhibitors for the target enzyme. Target specificity is crucial in drug development, as it minimizes the potential for off-target effects and improves the therapeutic index of the drug (Mizuno et al., 2003). Several methods can be employed to enhance the specificity of natural compounds. Structure-based design uses computer modeling to develop compounds that specifically bind to target enzymes (Read et al., 2001b), with this approach being especially effective when the three-dimensional structure of the enzyme is already known (Schmidt et al., 2014). Molecular docking, employing computer algorithms, predicts compound binding to the intended enzyme (Abdolmaleki et al., 2017). To improve specificity, one strategy involves identifying compounds that favor the target enzyme while having minimal impact on off-target enzymes (Xu et al., 2021). High-throughput screening on compound libraries is another approach (Mayr and Bojanic, 2009), testing a large number of compounds to identify those that work well with the target enzyme while suppressing other enzymes (Bachovchin et al., 2009).

### 6.6 Toxicity

Toxicity concerns are prevalent in drug development, especially when working with natural compounds, as they have the potential to harm healthy cells (Ali Abdalla et al., 2022). One approach to reduce toxicity is through the use of prodrugs and proper carriers. Various techniques, including conjugation, can decrease toxicity by refining pharmacokinetic features, enhancing precision in targeting disease cells, and minimizing risks to healthy cells (Ma et al., 2019).

Combining natural compounds with suitable carriers has led to successful cancer treatments, such as antibody-drug conjugates, which function by attaching a toxin to an antibody that specifically targets cancer cells, delivering the toxin directly to the intended site (Dosio et al., 2011). This targeted delivery system not only reduces toxicity in healthy cells but also enhances the cancer cell specificity of natural compounds (Puthenveetil et al., 2016). An example of this progressive method involves combining a special peptide with curcumin, which specifically targets EGFR and has a more pronounced impact on suppressing breast cancer cells. The peptide–curcumin conjugate directs its attention toward EGFR-positive cancer cells, hindering their growth without significantly affecting healthy cells (Jin et al., 2017).

### 6.7 Intellectual property

The development of natural compounds as LDH inhibitors faces a major obstacle in the form of intellectual property challenges. Given that these compounds are found in nature, they cannot be patented, preventing companies from obtaining exclusive rights to their use (Harrison, 2014). To address this issue, one possible solution is to create modified medications with unique characteristics (Kirschning et al., 2007). These modified versions can be patented as fresh innovations, protecting the investment in drug development while still making the original natural compound available for other purposes. Additionally, exploring innovative delivery systems can enhance the potency of natural compounds and reduce their harmful effects. Overcoming the complexities of intellectual property allows companies to secure exclusive rights to the use of delivery technologies, facilitating the development of effective and commercially viable natural compound–based drugs.

### 6.8 Clinical trials

Clinical trials play a critical role in evaluating the safety and efficacy of drug candidates before their approval for human use (Deore et al., 2019). Extensive preclinical and clinical testing is essential for natural compounds being developed as LDH inhibitors to assess their safety and efficacy in humans. Clinical trials for LDH inhibitors present unique challenges but are vital for evaluating the safety and efficacy of these compounds in humans. Developing LDH inhibitors for cancer treatment requires specific patient populations and endpoints for clinical trials. Currently, no United States Food and Drug Administration–approved LDH inhibitors for cancer treatment exist, and their development necessitates comprehensive clinical testing to assess efficacy and safety. Some natural compounds, such as gossypol and galloflavin, have shown potential as LDH inhibitors in preclinical studies, demonstrating both LDH activity inhibition and anti-cancer properties (Cui et al., 2017; Rani and Kumar, 2017). LDHB is associated with an aggressive cancer phenotype, and there are studies aimed at identifying and clinically applying selective inhibitors for LDHB (McCleland et al., 2012; Shibata et al., 2021). In a study of clinical samples from colorectal cancer patients, a significant correlation was observed between MYC expression and the expression of multiple metabolic genes, accompanied by elevated LDHB levels, while LDHA levels remained unchanged (Satoh et al., 2017). The approach of promoting cancer cell necrosis through the inhibition of lactate transport is presently being employed in initial clinical trials as a potential cancer treatment strategy, utilizing the selective MCT-1 inhibitor known as AZD3965 (Beloueche-Babari et al., 2020).

No existing LDH inhibitors have yet shown clinically significant effects, but research is underway to discover new ones using computer-based structure-based virtual screening methods (Di Magno et al., 2022). However, the clinical advancement of these compounds faces challenges, including effectiveness, toxicity, specificity, and bioavailability. These obstacles underscore the importance of thorough preclinical and clinical testing in advancing natural compounds as LDH inhibitors.

## 7 Combination therapy: Natural compounds and LDH inhibitors

Natural compounds and LDH inhibitors show promise as therapeutic agents for various conditions, including cancer, inflammation, and metabolic disorders (Granchi et al., 2010; Jacobs et al., 2017). However, using these agents alone may not yield optimal treatment results in certain scenarios. Employing combination therapy, which involves multiple drugs with complementary mechanisms of action, could offer a more effective treatment option (Luo et al., 2017; Cummings et al., 2019). By combining natural compounds with LDH inhibitors, treatment efficacy can be enhanced while minimizing the risk of toxicity (Augoff et al., 2015). Natural compounds and LDH inhibitors can target different pathways relevant to disease progression, complementing each other and improving therapeutic outcomes (Gallagher et al., 2017). Additionally, using natural compounds may mitigate certain drawbacks of LDH inhibitors, such as potential toxicity and restricted specificity to targets (Fiume et al., 2014).

Several studies have investigated the combination of existing therapeutic agents and LDH inhibitors for cancer treatment (Gallo et al., 2015). For instance, in breast cancer cells, the combination of the LDH inhibitor gallic acid with the phenolic compound curcumin induces apoptosis through glutathione reduction, ROS induction, and mitochondrial dysfunction (Moghtaderi et al., 2018). In androgen-dependent prostate cancer cells (LAPC-4 and LNCaP), the combination of quercetin and arctigenin significantly inhibited the PI3K/Akt pathway, resulting in enhanced anti-proliferative effects (Wang et al., 2015c).

In glioblastoma treatment, quercetin has shown the ability to augment the effects of drugs, including temozolomide, a DNA-methylating agent. When administered together, quercetin and temozolomide induced apoptosis in T98G cells by promoting cytochrome c release and reducing mitochondrial membrane potential (∆Ψm) (Jakubowicz-Gil et al., 2013). Moreover, quercetin enhances the sensitivity of glioblastoma cells to temozolomide by inhibiting the expression of heat shock protein 27, a molecular chaperone involved in apoptosis regulation (Sang et al., 2014). Combining curcumin and quercetin provides potent protection against myocardial toxicity induced by ischemia-reperfusion injury in rats (Chakraborty et al., 2018). In glioma stem-like cells, the combination of EGCG and temozolomide exerted inhibitory effects on neurosphere formation and cell migration (Zhang et al., 2015b), affecting migration and adhesion processes (Pilorget et al., 2003).

In a rat study, the combination of resveratrol and syringic acid showed synergistic protection against cardiotoxicity by reducing nuclear factor kappa B activation and lowering tumor necrosis factor alpha levels (Shaik et al., 2020). The combined treatment of resveratrol and paclitaxel in DBTRG glioblastoma cells led to increased apoptosis marker levels, caspase 3 activity, Ca^2+^ fluorescence intensity, ROS levels, mitochondrial function, mitochondrial membrane depolarization, and TRPM2 current density, resulting in reduced cell viability (Øztürk et al., 2019).

The mechanism underlying the synergistic effects of combining natural compounds and LDH inhibitors in therapy is not fully understood. Nevertheless, natural compounds may enhance the anti-tumor effects of LDH inhibitors by influencing the tumor microenvironment and promoting tumor cell demise (Kooshki et al., 2022). Through LDH inhibition, natural compounds may heighten the susceptibility of tumor cells, decreasing their energy metabolism and increasing their dependence on glycolysis (Gao and Chen, 2015). Combining natural compounds and LDH inhibitors can overcome the limitations of each agent used alone by reducing their concentration in the body. This approach alleviates the toxicity of LDH inhibitors and enhances the specificity of targeting tumor cells (Akbari et al., 2022).

## 8 Conclusion

Natural compounds such as LDH inhibitors have shown great potential for treating various diseases, including cancer, cardiovascular diseases, and neurodegenerative diseases (Leuci et al., 2020; Mohd Sairazi and Sirajudeen, 2020). Despite notable challenges that must be addressed, preclinical studies have demonstrated the safety and effectiveness of these compounds in this inhibitory role (Rani and Kumar, 2016; 2017). Combining natural compounds with existing LDH inhibitors has also shown promise in improving therapeutic outcomes and reducing toxicity (Augoff et al., 2015). These findings suggest that developing natural compounds as LDH inhibitors could lead to new treatments for several diseases. Indeed, natural compounds could be used as monotherapy for less aggressive tumors, as adjuvants to enhance chemotherapy effectiveness, and as complementary therapy for cardiovascular diseases and neurodegenerative diseases.

However, before using natural compounds as LDH inhibitors in clinical settings, several issues need to be addressed. Improving natural compound bioavailability and pharmacokinetics is essential to ensure optimal efficacy (Nowak et al., 2019). This can be achieved through the use of delivery systems, such as nanoparticles or liposomes, or by modifying the chemical structure of natural compounds (Aqil et al., 2013; Gunasekaran et al., 2014). Additionally, more research is needed to understand the molecular mechanisms through which natural compounds act as LDH inhibitors. Identifying specific targets and pathways influenced by natural compounds could lead to more effective and targeted therapies (Dutta et al., 2019). Furthermore, thorough clinical trials are necessary to assess the safety and toxicity of natural compounds. Despite being generally considered safe, some natural compounds may have undesirable side effects or interact with other medications (Scott and Elmer, 2002; Butler, 2008). Therefore, before using natural compounds in clinical settings, their safety and toxicity must be carefully evaluated.

Despite these challenges, the potential clinical uses of natural compounds as LDH inhibitors are promising. The development of natural compounds as LDH inhibitors offers hope for patients with various diseases. They can be used as monotherapy or adjuvants in cancer treatment (Rani and Kumar, 2016; Memariani et al., 2021), as a complementary therapy in cardiovascular disease, or to halt the progression of atherosclerosis (Zhang et al., 2021a), and to reduce neuroinflammation and oxidative stress as key pathological features in neurodegenerative diseases (Chen et al., 2020). In conclusion, natural compounds have shown promise as LDH inhibitors for treating various diseases. Although challenges remain in their clinical application, further research and development could pave the way for novel treatments, offering renewed hope to patients suffering from diverse diseases.
